# Research on placebo analgesia is relevant to clinical practice

**DOI:** 10.1186/2045-709X-22-6

**Published:** 2014-02-03

**Authors:** Charles W Gay, Mark D Bishop

**Affiliations:** 1 Rehabilitation Science Doctoral Student, University of Florida, Gainesville, Florida, USA; 2 Associate Professor, Department of Physical Therapy, University of Florida, Gainesville, Florida, USA; 3 Center for Pain Research & Behavioral Health, University of Florida, Gainesville, Florida, USA

**Keywords:** Placebo, Endogenous pain modulation, Placebo response

## Abstract

Over the decades, research into placebo responses has shed light onto several endogenous (i.e. produced from within) mechanisms underlying modulation of pain perception initiated after the administration of inert substances (i.e. placebos). Chiropractors and manual therapists should embrace analgesic-placebo-research in an attempt to maximize clinical benefit. Historical views that placebo responses are fake, passive, undesirable, and require deception and therefore should be minimized and avoided in clinical practice are outdated. Further, statements that contend the placebo response represents a single mechanism are overly simplistic. This commentary will discuss research that shows that there are several active biological processes underlying modulation of pain perception involved in placebo analgesia and its counterpart nocebo hyperalgesia. We contend that it is highly likely that, to some extent, all of these biological processes are engaged, in varying degrees, following all interventions and represent endogenous pain modulating processes. Failure, of chiropractors and manual therapists, to embrace a more contemporary view of analgesic-placebo-research serves as a barrier to transferring knowledge into clinical practice and represents a missed opportunity to improve the delivery of current treatments.

## Background

Evidence-based medicine deals with the use of current best evidence in clinical decision-making and other aspects of patient care. The assumption that simply having the evidence available or providing authoritative clinical practice guidelines will result in practice change and improved patient outcomes is incorrect [1,2]. A number of physician behaviors have been identified that act as barriers to translating evidence into clinical practice [3]. Two domains of those barriers are physician’s knowledge and attitudes. This commentary addresses knowledge awareness and outdated attitudes surrounding the clinical improvement seen following the administration of a placebo. By addressing these potential barriers chiropractors and manual therapists may be more open to analgesic-placebo-research and its counterpart hyperalgesia-nocebo-research. In turn, clinical practice may be influenced by knowledge gained from this field, applying evidence to decision-making and the context in which interventions are delivered with a goal of improving patient outcomes.

### Main text

Misconceptions about placebo responses continue in the general public and among healthcare professionals [4,5]. There has been a push for healthcare professionals to re-think what a placebo response represents and the applicability of knowledge gained from placebo-analgesia-research that can be applied ethically in clinical practice [6-12]. Rethinking placebo and nocebo responses as endogenous modulatory mechanisms broadens the focus of care beyond just the intervention to include the context in which interventions are delivered. Historical views that placebo responses represent fake, passive, and undesirable results; require deception; and should be minimized and avoided in clinical practice continue today among healthcare providers. Further, the placebo response has been described as a single mechanism through which an intervention may induce a positive therapeutic outcome [9]. Harboring such views may bias manual therapy practitioners away from valuable clinical evidence that may influence their clinical decision making. This commentary presents research that opposes these views.

Changes in the perceived intensity of pain are often attributed to a variety of factors lumped into three board categories: (1) condition related factors, (2) the specific effects of treatment, and/or (3) treatment contextual effects [13]. Condition related factors include the natural course of the condition and regression to the mean. Specific treatment effects are the unique effects associated with the 'active’ ingredient of the treatment. Treatment context includes a host of signs, symbols and physician interactions that convey information to the individual. In recent years, researchers have used placebo-analgesia and its negative equivalent, nocebo-hyperalgesia to investigate the neuronal underpinnings of treatment contextual effects. Although, these three categories are practical and useful, they can create a misconception that there are unique biological pathways that do not overlap or interact underlying the categories. This misconception is highlighted by the notion that a predictable estimate of change in pain perception can be determined simply by a treatment’s active biological properties.

For example, findings from one study significantly challenge the convention that an active biological agent produces a consistent therapeutic effect within an individual. By convention, researchers acknowledge that the active biological agent in a drug will vary across people, depending on a multitude of personal factors. However, what is not acknowledged so frequently is that within one individual, the therapeutic effect can significantly vary. By experimentally manipulating an individual’s expectation through instructional sets, it was shown that the therapeutic effect of the active ingredient of remifentanil, a potent synthetic μ-opioid agonist, can be significantly modulated [14]. The study manipulated subjects’ expectations by instructing them that remifentanil is a widely used opioid that relieves pain when infused intravenously, but can worsen pain when the infusion ceases [14]. Hidden administration of the drug produced less perceived pain than baseline, open administration of the drug produced significantly more hypoalgesia than hidden administration and when the drug was continuously administered but the subjects were told it had stopped, the perception of pain retuned to baseline values [14]. The estimated therapeutic effect size of the active ingredient in remifentanil spanned from no effect to a moderate effect, depending on the expectancy created by the instructional set.

Embedded in every pain-relieving treatment are contextual effects. Physician interactions, signs and symbols convey information to the individual with the potential for producing therapeutic and counter-therapeutic responses (ie placebo and nocebo responses respectively) [15]. The estimated therapeutic effect of treatment context following the administration of an inert substance has been shown to be substantial, albeit variable [15-21]. One factor that is not causing the therapeutic effect is the inert substance (ie placebo). Therefore, placebo-analgesia-research provides valuable information about the context in which interventions are delivered. Research into principle mediators of the contextual effects suggest expectancy, desire for a positive outcome and classical conditioning account for a significant portion of the variance in contextual effects [22-24]. Chiropractors and manual therapists should be aware that the context in which they deliver their interventions is as important as the intervention they are giving. Further evidence suggesting the effectiveness of a manual therapy intervention is influenced by patient’s expectation comes from a study published by Bialosky et al. [25]. The study showed that by using different instructional sets to positively, negatively and neutrally influence the subjects expectation, the effect of spinal manipulation on a dynamic pain sensitivity measure (temporal summation of second pain) varied [25].

Placebo-analgesia-research has also shown that deception is not integral to inducing a placebo response. One study administered open labeled placebos with patient education that described an active biological pathway for symptom improvement [26]. In this study, the investigators told irritable bowel syndrome (IBS) subjects, that they would be randomized to receive either a placebo sugar pill or no treatment. Subjects receiving the placebo pill were told placebos have been found to produce clinically effective results though a mind-body connection and that by taking the pill, the subjects would be harnessing their own recuperative powers. Greater clinical improvement was found in the placebo group compared to the no-treatment group [26]. Another study examined the repeatability of contextual effects after subjects were told they received an inert substance. In this study, individuals, who received inert pain-relieving cream and experienced pain reductions, were then told they had received a placebo cream. On a subsequent visit those same individuals were again able to experience pain relief a second time using an inert cream [27]. Both of these studies challenge deception’s role as a necessary component of the context in which an inert treatment is delivered that produces a positive therapeutic effect.

Finally, placebo-analgesia-research has shed light onto neural mechanisms underlying endogenous pain modulation. Cortical networks involved in processing and modulating the pain experience have been identified and continue to be reinvestigated, redefined and undergo reconceptualization. Activity in these cortical networks influence the perception of pain and modulation can be inhibitory or facilitatory. Research has identified various neurotransmitters including opioids, cannabinoids, dopamines and cholecystokinins, that are used within these cortical networks [28-31]. Attributing clinical benefit following the administration of an inert substance to a single mechanism, or terming it a placebo mechanism fails to account for the numerous neural and other biological pathways involved in producing and influencing the perception of pain [32,33].

## Conclusion

Over the decades, research into placebo responses has shed light onto several endogenous (i.e. produced from within) mechanisms underlying modulation of pain perception initiated by the administration of inert substances (i.e. placebos/nocebos). In addition, this growing body of work emphasizes the need for chiropractors’ and manual therapists’ to be more alert to, and embrace, 'psychologically informed practice’; that is, practice in which recognition of neural mechanisms is equally as important as identification of structural pathology. Chiropractors and manual therapists should embrace analgesic-placebo-research in an attempt to maximize endogenous pain relieving mechanisms to produce maximum clinical benefit. Historical views that placebo responses are fake, passive, undesirable, and require deception and therefore should be minimized and avoided in clinical practice are outdated. Further, statements that contend the placebo response represents a single mechanism are too simplistic. Instead, in this commentary the authors discussed how several active biological processes underlie analgesic-placebo responses. We contend that it is highly likely that to some extent all of these biological processes, which represent endogenous pain relieving processes, are engaged to various degrees following all interventions that produce a positive clinical outcome.

Failure to embrace a more contemporary view of analgesic-placebo-research may negatively bias chiropractors’ and manual therapists’ opinions about the potential clinical value of results emerging from this field. These biases serve as a barrier to successfully translating potential benefits for patients into clinical practice in an ethical manner. Analgesic-placebo-research provides insight on how to improve the delivery of current treatments by optimizing clinical benefit and matching the right treatment to the right spinal pain patient at the right time.

## Competing interests

The authors of this manuscript attest there are no conflicts of interest to report.
